# Association of maternal serum 25-hydroxyvitamin D concentrations in second and third trimester with risk of macrosomia

**DOI:** 10.1038/s41598-018-24534-5

**Published:** 2018-04-18

**Authors:** Juan Wen, Congli Kang, Jiaan Wang, Xianwei Cui, Qin Hong, Xingyun Wang, Lijun Zhu, Pengfei Xu, Ziyi Fu, Lianghui You, Xing Wang, Chenbo Ji, Xirong Guo

**Affiliations:** 1 Nanjing Maternity and Child Health Care Institute, The Affiliated Obstetrics and Gynecology Hospital of Nanjing Medical University (Nanjing Maternity and Child Health Care Hospital), Nanjing, China; 2 Department of Children Health Care, The Affiliated Obstetrics and Gynecology Hospital of Nanjing Medical University (Nanjing Maternity and Child Health Care Hospital), Nanjing, China; 3 State key Laboratory of Reproductive Medicine, The Affiliated Obstetrics and Gynecology Hospital of Nanjing Medical University (Nanjing Maternity and Child Health Care Hospital), Nanjing, China; 4 Department of clinical laboratory, People’s Hospital of Rizhao, Rizhao, China

## Abstract

Whether the maternal vitamin D deficiency is associated with infant birth weight is still an argument. Here, we performed a nested case-control study (545 women who subsequently delivered infant with macrosomia and 1090 controls) to evaluate the association of the maternal serum 25-hydroxyvitamin D [25(OH)D] concentrations with risk of macrosomia. We measured the serum 25(OH)D concentrations by enzyme immunoassays. Logistic regression analysis, receiver-operator characteristic curve analysis and graphical nomogram were used for the statistical analyses. Among women who delivered infant with macrosomia, 71.2% of the women had serum 25(OH)D concentrations <50.0 nmol/L compared with 61.1% of the control women (*P* < 0.001). For women with concentrations <50.0 nmol/L, they had a 33% increased risk of macrosomia compared with women whose 25(OH)D ranged from 50.0 to 74.9 nmol/L. The risk of macrosomia was significantly increased with the decreasing concentrations of serum 25(OH)D in a dose-dependent manner (*P* for trend = 0.001). We also observed a threshold for 25(OH)D of 50.0 nmol/L for delivering infant with macrosomia and a predictive accuracy of the 25(OH)D concentrations included panel, with an area under the ROC curve of 0.712 for delivering infant with macrosomia. In conclusion, maternal serum 25(OH)D <50.0 nmol/L is associated with delivering a macrosomic infant, and vitamin D deficiency should be monitored in pregnant women.

## Introduction

Birth weight (BW) is an essential indicator of newborns’ nutritional and developmental status and plays an important role in infant survival, childhood development and adult cardio-metabolic diseases^1^. Abnormal BW can be divided into two categories, that is low birth weight (LBW) (birth weight <2.5 kg) and macrosomia (birth weight ≥ 4.0 kg), both of which are strongly associated with a variety of short- and long-term developmental and health problems^2,3^. LBW is one of the major causes of neonatal mortality and child morbidity, and its incidence varies from 6.1% to 11.0%^4,5^. It has also been linked to an increased risk of growth retardation and chronic diseases later in life, such as metabolic disorders and heart disease^6^. Of the LBW infants, small-for-gestational age (SGA) newborns have attracted much attention due to their high prevalence and debilitating consequences^7–9^. On the other hand, the incidence of macrosomia worldwide in recent decades was 4.7–13.1%^5,10^. Macrosomia is characterized by asymmetric growth of the abdominal circumference and an excess of fat accumulation^11^. Studies have shown that macrosomia is related to an increased risk of caesarean birth, delivery complications, and subsequent obesity, metabolic diseases and certain cancers^12^. Thus, investigating abnormal BW and its risk factors has important public health implications.

Research has shown that gestational weeks at birth, pre-pregnant body mass index (BMI), gestational weight gain, fetus gender, birth season, state of gestational diabetes and genetic factors could influence BW^13,14^. Whether maternal vitamin D deficiency is associated with infant BW remains a topic of debate. Due to fetal growth needs, inadequate vitamin D intake and limited sunlight exposure, vitamin D deficiency is very common in pregnant women^15^. The association of maternal vitamin D levels with fetal growth has been investigated by numerous observational studies and randomized controlled trials, more of which focused on infant BW and SGA and rarely considered macrosomia^13,14,16–19^. 25-hydroxyvitamin D [25(OH)D], an indicator of vitamin D levels, was measured in maternal serum or cord blood in most studies. Some studies provided evidence that there is an inverted U-shaped relation between 25(OH)D concentrations and fetal growth^14,16^ and suggested that low 25(OH)D concentrations are associated with a higher risk of SGA^20,21^. However, other studies did not find any evidence of the association^13,18^ or reported an increased risk of macrosomia for pregnant woman with low 25(OH)D concentrations^19,22^. The conflicting findings may be due to variations in the study designs, including sample sizes, race, gestational weeks of sampling, cut-offs and quantification methods for 25(OH)D, adjusting for critical confounders and genetic factors.

In our previous large cohort study, we found that women with 25(OH)D <37.5 nmol/L had infants with higher BW in a linear regression model^22^. To further evaluate the relationship between maternal vitamin D deficiency and the risk of macrosomia, we performed a nested case-control study in a 1:2 ratio, including 545 women who subsequently delivered infant with macrosomia and 1090 women who delivered neonate of normal weight (as controls). Furthermore, we evaluated the threshold of 25(OH)D for macrosomia and the performance of low 25(OH)D in predicting delivering macrosomia.

## Results

**Figure 1:**
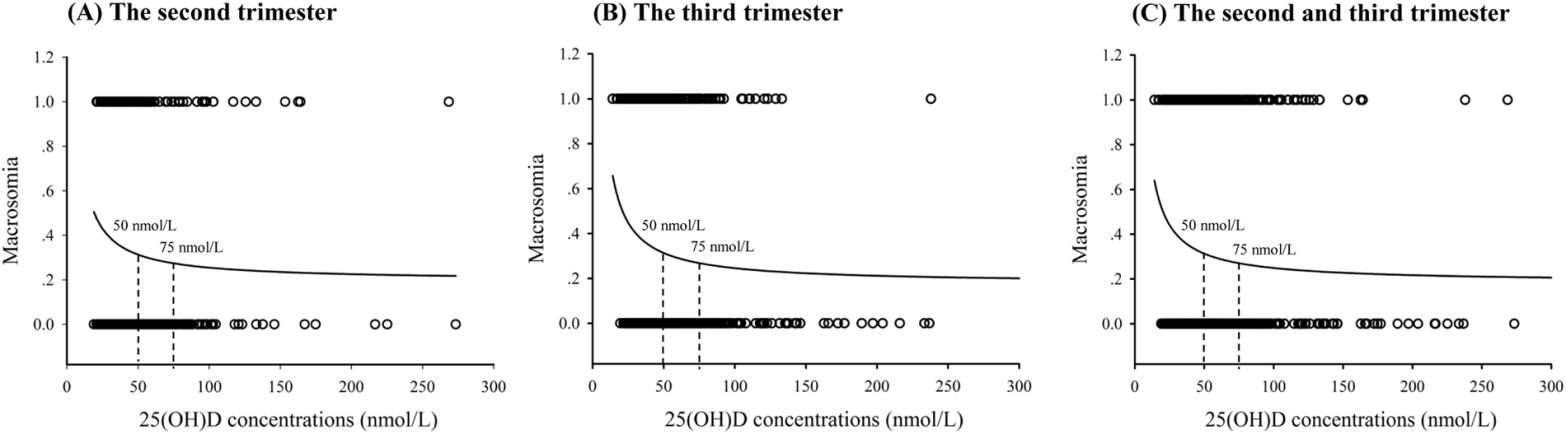
The relationship between maternal 25(OH)D and macrosomia. A nonlinear relationship between the serum 25(OH)D and macrosomia was observed. (**A**) For women in the second trimester; (**B**) For women in the third trimester; (**C**) For all the women. 25(OH)D, 25-hydroxyvitamin D.

We successfully analysed the serum 25(OH)D concentrations from all 1635 samples (545 women who delivered infant with macrosomia and 1090 controls). There were no significant differences in the distribution of maternal age and birthplace between the groups. However, women who delivered infant with macrosomia were more likely to have higher intrapartum BMI and more gestational weeks at birth, were more likely to have gestational diabetes, and were less likely to be nulliparae as compared with controls (all *P* < 0.05). The rate of male fetus was significantly higher in cases than in controls (*P* < 0.001) (Table 1). The maternal serum 25(OH)D concentrations were lower in women who delivered infant with macrosomia [median (IQR), women delivered macrosomia vs. controls: 41.4 (34.3, 52.5) vs. 45.0 (36.2, 59.8) nmol/L, *P* < 0.001]. Among women who delivered infant with macrosomia, 71.2% of the women had serum concentrations <50.0 nmol/L, compared with 61.1% of the control women (*P* < 0.001) (Table 1). In addition, there was a negative correlation between birth weight and the 25(OH)D concentrations (r = −0.071, *P* = 0.004). As shown in Fig. 1, there was a nonlinear relationship between serum 25(OH)D and macrosomia, with a threshold for 25(OH)D of 50.0 nmol/L for macrosomia.

**Table 1:** Maternal characteristics and serum 25(OH)D concentrations between cases and controls.

Maternal characteristics	Women who delivered infant with macrosomia	Controls	*P* ^†^
	(n = 545)	(n = 1090)	
Maternal age (year)^*^	28.8 ± 3.4	29.2 ± 9.1	0.277
Birthplace of Jiangsu province [n (%)]	525 (96.3)	1038 (95.2)	0.353
Intrapartum BMI (kg/m^2^)^*^	29.0 ± 3.4	26.7 ± 3.0	<0.001
Gestational weeks at birth^*^	39.5 ± 1.0	39.1 ± 1.1	<0.001
Sampling gestational weeks^‡^	28 (27, 30)	28 (27, 29)	0.443
Fetus gender (Male) [n (%)]	362 (66.4)	534 (49.0)	<0.001
Birth weight (g)^*^	4142.8 ± 187.2	3362.8 ± 321.2	<0.001
Gestational diabetes [n (%)]	169 (31.0)	286 (26.2)	0.042
Nulliparae [n (%)]	497 (91.2)	1029 (94.4)	0.014
Having abnormal pregnancy history [n (%)]	88 (16.1)	178 (16.3)	0.925
Sampling season [n (%)]			0.919
Spring	140 (25.7)	282 (25.9)	
Summer	166 (30.5)	337 (30.9)	
Autumn	126 (23.1)	236 (21.7)	
Winter	113 (20.7)	235 (21.6)	
25(OH)D (nmol/L)^‡^	41.4 (34.3, 52.5)	45.0 (36.2, 59.8)	<0.001
25(OH)D [n (%)]			0.001
<25.0 nmol/L	21 (3.9)	27 (2.5)	
25.0–37.4 nmol/L	173 (31.7)	291 (26.7)	
37.5–49.9 nmol/L	194 (35.6)	348 (31.9)	
50.0–74.9 nmol/L	115 (21.1)	281 (25.8)	
>75 nmol/L	42 (7.7)	143 (13.1)	

^*^Mean ± SD; ^†^*P* < 0.05 (chi-square test, *t* test, or Mann-Whitney test as appropriate).

^‡^Median (IQR); 25(OH)D, 25-hydroxyvitamin D; BMI, body mass index.

Logistic regression analyses showed that women with 25(OH)D concentrations <25.0 nmol/L, from 25.0 to 37.4 nmol/L and from 37.5 to 49.9 nmol/L all had an increased risk of macrosomia compared with women who had concentrations ranging from 50.0 to 74.9 nmol/L. In addition, the risk of macrosomia was significantly increased with the decreasing concentrations of serum 25(OH)D in a dose-dependent manner (*P* for trend = 0.001). Women with concentrations <50.0 nmol/L had an increased risk of macrosomia (adjusted OR = 1.33, 95% CI = 1.01–1.74), after adjusting for confounders (Table 2). The association of 25(OH)D concentrations with the risk of macrosomia was also evaluated after stratifying by maternal age, intrapartum BMI, gestational weeks at birth, fetus gender, gestational diabetes status, parity, sampling trimester, abnormal pregnancy history and sampling season (Table 2). Similar association strengths were shown between most subgroups (*P* > 0.05 for heterogeneity test). Interestingly, a stronger effect of the 25(OH)D concentrations <50.0 nmol/L on macrosomia risk was observed among pregnant woman with a male fetus (adjusted OR = 1.74, 95% CI = 1.21–2.49) compared with that observed in women with a female fetus (adjusted OR = 0.98, 95% CI = 0.65–1.48) (*P* = 0.040 for heterogeneity test). A significantly multiplicative interaction between the serum 25(OH)D concentrations and fetus gender on macrosomia risk was detected by further interactive analysis (*P* = 0.031) (Table 3). Crossover analysis suggested that “serum 25(OH)D concentrations <50.0 nmol/L” with “male fetus” had a significant risk effect (adjusted OR = 2.23, 95%CI = 1.51–3.28, *P* < 0.001) for macrosomia, when compared with the combination of “serum 25(OH)D concentrations ≥50.0 nmol/L” with “female fetus” (Table 3).

**Table 2:** The associations between maternal serum 25(OH)D concentrations and risk of macrosomia and stratified analyses on the associations.

Variables	Univariate						Multivariate					
	25(OH)D (nmol/L)						25(OH)D (nmol/L)					
	<25.0	25.0–37.4	37.5–49.9	<50.0	50.0–74.9	>75	<25.0	25.0–37.4	37.5–49.9	<50.0	50.0–74.9	>75
All women	1.90 (1.03–3.50)	1.45 (1.09–1.94)	1.36 (1.03–1.80)	1.42 (1.11–1.83)	1.00 (ref)	0.72 (0.48–1.08)	1.59 (0.82–3.08)	1.36 (1.00–1.85)	1.27 (0.94–1.72)	1.33 (1.01–1.74)	1.00 (ref)	0.66 (0.43–1.03)
**Maternal age** (**year)**												
<30	1.98 (0.93–4.24)	1.44 (1.00–2.08)	1.41 (0.99–2.01)	1.45 (1.05–1.99)	1.00 (ref)	0.87 (0.52–1.46)	1.63 (0.71–3.75)	1.32 (0.90–1.95)	1.34 (0.92–1.96)	1.34 (0.95–1.89)	1.00 (ref)	0.82 (0.47–1.43)
30~	1.76 (0.63–4.92)	1.48 (0.92–2.36)	1.32 (0.84–2.09)	1.41 (0.93–2.12)	1.00 (ref)	0.53 (0.27–1.03)	1.40 (0.46–4.31)	1.46 (0.87–2.43)	1.16 (0.70–1.91)	1.31 (0.84–2.05)	1.00 (ref)	0.44 (0.21–0.92)
**Intrapartum BMI** (**kg/m2)**												
<30	1.57 (0.73–3.38)	1.52 (1.09–2.13)	1.31 (0.94–1.83)	1.42 (1.05–1.90)	1.00 (ref)	0.79 (0.50–1.27)	1.53 (0.69–3.42)	1.43 (1.01–2.03)	1.28 (0.91–1.80)	1.36 (1.00–1.85)	1.00 (ref)	0.77 (0.47–1.25)
30~	1.72 (0.53–5.58)	1.09 (0.58–2.05)	1.26 (0.69–2.30)	1.21 (0.70–2.09)	1.00 (ref)	0.45 (0.18–1.12)	1.49 (0.43–5.20)	1.12 (0.57–2.22)	1.18 (0.62–2.26)	1.16 (0.64–2.09)	1.00 (ref)	0.38 (0.14–1.01)
**Gestational weeks at birth**												
<40	1.87 (0.79–4.44)	1.51 (1.00–2.27)	1.39 (0.93–2.06)	1.46 (1.02–2.08)	1.00 (ref)	0.69 (0.38–1.26)	1.54 (0.61–3.91)	1.43 (0.93–2.21)	1.26 (0.83–1.92)	1.36 (0.93–1.99)	1.00 (ref)	0.65 (0.34–1.25)
40~	1.89 (0.77–4.64)	1.36 (0.90–2.05)	1.38 (0.92–2.08)	1.39 (0.97–2.00)	1.00 (ref)	0.72 (0.41–1.27)	1.47 (0.55–3.89)	1.27 (0.82–1.98)	1.25 (0.81–1.93)	1.27 (0.86–1.88)	1.00 (ref)	0.63 (0.34–1.14)
**Fetus gender**												
Male	3.94 (1.74–8.94)	1.57 (1.07–2.30)	1.62 (1.12–2.35)	1.67 (1.19–2.33)	1.00 (ref)	0.99 (0.60–1.65)	3.62 (1.51–8.69)	1.64 (1.09–2.48)	1.69 (1.14–2.52)	1.74 (1.21–2.49)	1.00 (ref)	0.97 (0.56–1.67)
Female	0.35 (0.08–1.56)	1.28 (0.82–2.00)	1.03 (0.66–1.59)	1.10 (0.75–1.62)	1.00 (ref)	0.36 (0.17–0.77)	0.27 (0.05–1.30)	1.11 (0.69–1.78)	0.91 (0.57–1.45)	0.98 (0.65–1.48)	1.00 (ref)	0.36 (0.16–0.80)
**Gestational diabetes**												
No	1.90 (0.91–3.98)	1.29 (0.92–1.80)	1.38 (1.00–1.90)	1.36 (1.02–1.81)	1.00 (ref)	0.59 (0.36–0.94)	1.53 (0.69–3.40)	1.17 (0.82–1.68)	1.22 (0.86–1.72)	1.21 (0.89–1.65)	1.00 (ref)	0.53 (0.32–0.88)
Yes	1.91 (0.64–5.72)	1.87 (1.05–3.33)	1.34 (0.76–2.36)	1.58 (0.94–2.67)	1.00 (ref)	1.45 (0.63–3.34)	1.81 (0.53–6.20)	2.11 (1.12–3.95)	1.54 (0.83–2.88)	1.79 (1.01–3.17)	1.00 (ref)	1.38 (0.55–3.44)
**Parity**												
Nulliparae	2.01 (1.07–3.77)	1.44 (1.07–1.94)	1.35 (1.01–1.80)	1.41 (1.09–1.83)	1.00 (ref)	0.71 (0.47–1.08)	1.76 (0.89–3.48)	1.37 (0.99–1.88)	1.26 (0.92–1.72)	1.33 (1.00–1.75)	1.00 (ref)	0.66 (0.42–1.03)
Multipara	0.83 (0.07–10.55)	1.49 (0.52–4.28)	1.50 (0.53–4.26)	1.46 (0.57–3.75)	1.00 (ref)	1.00 (0.19–5.22)	0.36 (0.02–6.57)	1.34 (0.39–4.60)	1.65 (0.47–5.83)	1.40 (0.46–4.31)	1.00 (ref)	0.59 (0.09–4.06)
**Sampling trimester**												
Second	1.50 (0.54–4.22)	2.00 (1.26–3.17)	2.05 (1.30–3.22)	2.00 (1.33–3.01)	1.00 (ref)	1.22 (0.64–2.34)	1.40 (0.47–4.22)	2.08 (1.27–3.42)	2.12 (1.30–3.45)	2.08 (1.34–3.22)	1.00 (ref)	1.27 (0.63–2.56)
Third	2.17 (1.00–4.73)	1.18 (0.81–1.70)	1.04 (0.73–1.49)	1.14 (0.83–1.57)	1.00 (ref)	0.51 (0.30–0.87)	1.62 (0.68–3.83)	1.06 (0.71–1.58)	0.94 (0.63–1.39)	1.01 (0.71–1.44)	1.00 (ref)	0.45 (0.25–0.79)
**Abnormal pregnancy history**												
No	2.51 (1.27–4.96)	1.50 (1.09–2.06)	1.44 (1.06–1.95)	1.50 (1.14–1.98)	1.00 (ref)	0.63 (0.39–1.00)	2.03 (0.97–4.25)	1.43 (1.02–2.01)	1.35 (0.97–1.87)	1.41 (1.04–1.90)	1.00 (ref)	0.55 (0.33–0.91)
Yes	0.54 (0.11–2.76)	1.26 (0.63–2.54)	1.05 (0.53–2.07)	1.10 (0.61–1.99)	1.00 (ref)	1.13 (0.48–2.67)	0.48 (0.08–2.86)	1.06 (0.49–2.27)	0.94 (0.44–2.02)	1.02 (0.53–1.99)	1.00 (ref)	1.03 (0.41–2.63)
**Sampling season**												
Spring/Winter	2.32 (1.10–4.89)	1.31 (0.87–1.98)	1.10 (0.72–1.67)	1.26 (0.87–1.82)	1.00 (ref)	0.67 (0.35–1.29)	1.85 (0.82–4.18)	1.16 (0.74–1.83)	0.98 (0.62–1.55)	1.10 (0.74–1.65)	1.00 (ref)	0.71 (0.35–1.44)
Summer/Autumn	1.02 (0.31–3.37)	1.61 (1.08–2.39)	1.62(1.11–2.36)	1.60 (1.13–2.25)	1.00 (ref)	0.76 (0.45–1.27)	0.76 (0.21–2.75)	1.56 (1.02–2.38)	1.56 (1.04–2.33)	1.54 (1.07–2.23)	1.00 (ref)	0.66 (0.38–1.16)

All values are ORs (95% CIs). Values were determined by using logistic regression. Adjusted values were adjusted for maternal age, birthplace, intrapartum BMI, gestational weeks at birth, fetus gender, status of gestational diabetes, parity, sampling trimester, abnormal pregnancy history and sampling season (excluded the stratified factor in each stratum). 25(OH)D, 25-hydroxyvitamin D.

**Table 3:** Interaction analyses on the serum 25(OH)D concentrations and fetus gender on risk of macrosomia.

Serum 25(OH)D concentrations	Fetus gender	Women who delivered infant with macrosomia (n = 545)	Controls (n = 1090)	OR (95%CI)	*P*
50~ nmol/L	Female	48 (9.5)	142 (15.0)	1.00	
<50 nmol/L	Female	126 (25.1)	339 (35.8)	0.94 (0.62–1.41)	0.756
50~ nmol/L	Male	67 (13.3)	139 (14.7)	1.31 (0.82–2.08)	0.257
<50 nmol/L	Male	262 (52.1)	326 (34.5)	2.23 (1.51–3.28)	<0.001
Interaction				P* = 0.031	

Logistic regression analyses adjusted for maternal age, birthplace, intrapartum BMI, gestational weeks at birth, status of gestational diabetes, parity, sampling trimester, abnormal pregnancy history and sampling season; *P value for multiplicative interaction. 25(OH)D, 25-hydroxyvitamin D.

**Figure 2:**
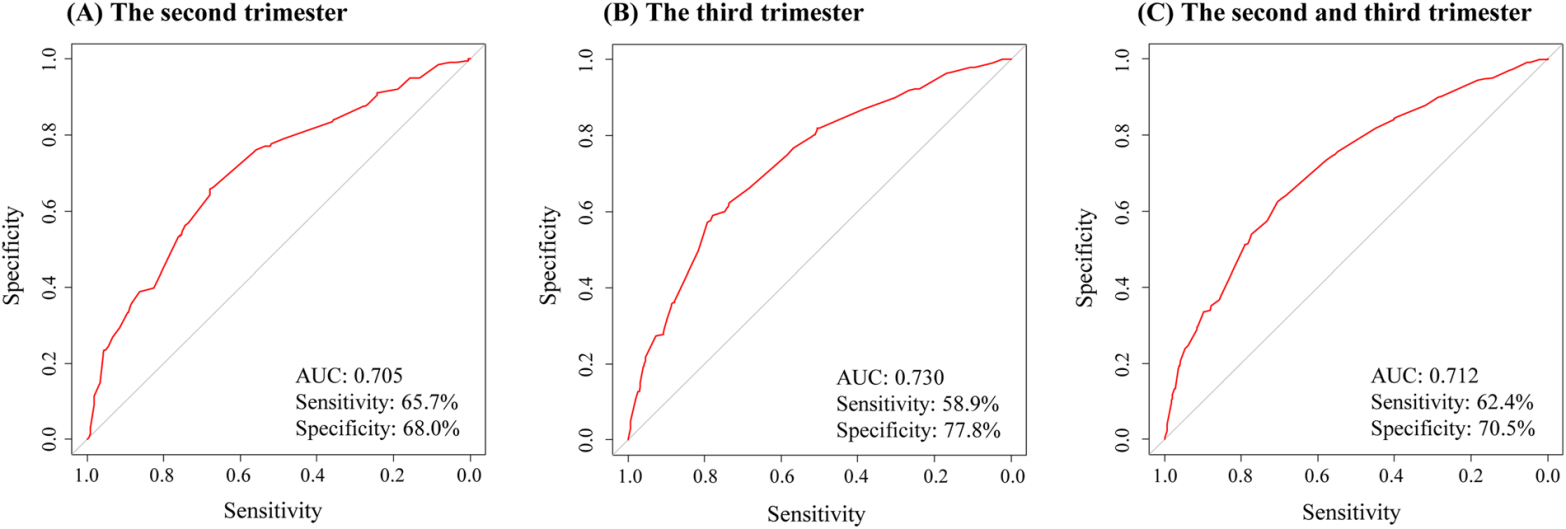
The discriminative ability of three panels between women who delivered infant with macrosomia and controls was evaluated by a ROC curve analysis. The panel included intrapartum BMI, gestational weeks at birth, fetus gender, parity and serum 25(OH)D; (**A**) For women in the second trimester; (**B**) For women in the third trimester; (**C**) For all the women. ROC, receiver-operator characteristic; BMI, body mass index; 25(OH)D, 25-hydroxyvitamin D.

**Figure 3:**
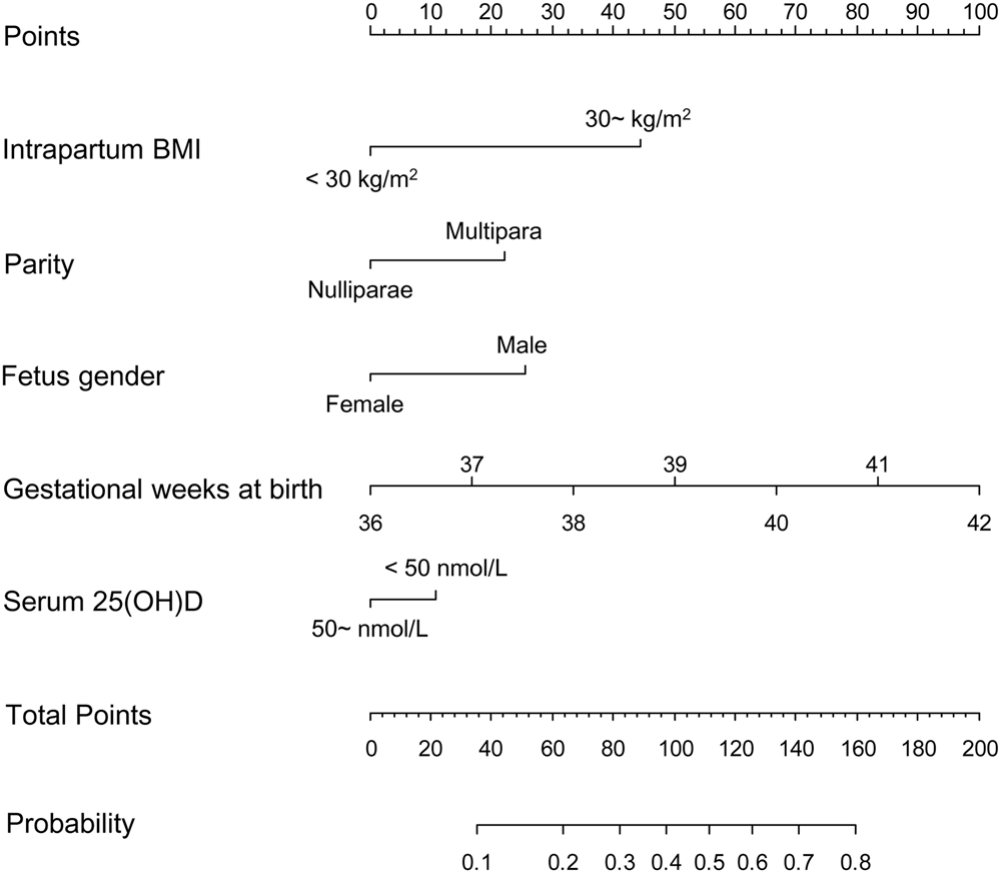
Predictive graphic nomogram for probability of delivering macrosomia. BMI, body mass index; 25(OH)D, 25-hydroxyvitamin D.

Then, we constructed risk prediction models to classify women who delivered macrosomia and controls. For all the women in the second and third trimester, after stepwise regression analysis, intrapartum BMI (30~ vs. <30 kg/m^2^), gestational weeks at birth, fetus gender (male vs. female), parity (multipara vs. nulliparae) and serum 25(OH)D (<50 vs. 50~ nmol/L) were entered into the final regression model (Table 4), suggesting that serum 25(OH)D <50.0 nmol/L is an independent risk factor for delivering infant with macrosomia (OR = 1.36, 95%CI = 1.04–1.78, *P* = 0.023). Then, we constructed a receiver-operator characteristic curve to assess the risk prediction performance of the entered variables for delivering infant with macrosomia (Fig. 2). For the panel including intrapartum BMI, gestational weeks at birth, fetus gender, parity and serum 25(OH)D, we observed a good predictive accuracy for delivering infant with macrosomia (sensitivity = 62.4%, specificity = 70.5%), with an area under the curve of 0.712. The Hosmer-Lemeshow *χ*^2^ was 10.29 (*P* = 0.173) for the panel, which gave no cause for concern over model fit or calibration. The graphical nomogram derived from the logistic regression is presented in Fig. 3. Each woman characteristic was aligned with the corresponding number of points on the uppermost point scale. After all characteristics were considered, the user summed all points and aligned the sum on the “total points” line with the predicted probability of delivering infant with macrosomia.

**Table 4:** Results of full model for macrosomia after stepwise regression analysis.

Variables	SE	Z	OR (95%CI)	*P*
Intrapartum BMI (30~ vs. <30 kg/m^2^)	0.52	8.98	3.61 (2.73–4.78)	<0.001
Gestational weeks at birth	0.10	7.98	1.62 (1.44–1.82)	<0.001
Fetus gender (male vs. female)	0.26	6.05	2.09 (1.65–2.66)	<0.001
Parity (multipara vs. nulliparae)	0.43	2.80	1.89 (1.21–2.95)	0.005
Serum 25(OH)D (<50 vs. 50~ nmol/L)	0.19	2.27	1.36 (1.04–1.78)	0.023

SE, standard error; Z, Z value; BMI, body mass index; 25(OH)D, 25-hydroxyvitamin D.

## Discussion

In this large nested case-control study conducted on macrosomia, we first found that the maternal serum 25(OH)D concentrations were significantly lower in women who subsequently delivered infant with macrosomia. Women with concentrations <50.0 nmol/L had a 33% increase in macrosomia risk compared with women with 25(OH)D ranging from 50.0 to 74.9 nmol/L. We also observed a threshold for 25(OH)D of 50.0 nmol/L for delivering infant with macrosomia and a good predictive accuracy of the 25(OH)D concentrations included panel. Further studies are warranted to validate and extend our findings. In general, our results suggested that maternal serum 25(OH)D <50.0 nmol/L may be an independent risk factor for delivering infant with macrosomia and that it should be monitored for high-risk pregnant women.

The prospective data collection, a relatively large sample size, random sampling, blinded analysis, and statistical adjustment in our study provided sufficient statistical power and convincing data. We concluded that low 25(OH)D concentrations in pregnancy were associated with an increased risk of macrosomia, which was contrary to the conclusions of most previous studies. In 2015, Zhu *et al*. measured the cord blood 25(OH)D concentrations in 1491 neonates in Hefei (China) and found that the neonates in the 4^th^ to 7^th^ deciles of cord blood 25(OH)D had significantly increased BW and decreased risk of SGA compared with neonates in the lowest decile^14^. A nested case-control study performed in white and black pregnant women showed that there was a U-shaped relation between serum 25(OH)D and SGA risk among white women, with the lowest risk at 60–80 nmol/L, but not among black women^16^. Another observational cohort conducted in 12 U.S. medical centres found that maternal serum 25(OH)D ≥37.5 nmol/L was associated with half the risk of SGA in the first trimester compared with 25(OH)D <37.5 nmol/L. However, no similar association in the second trimester was observed^17^. In contrast, Schneuer *et al*. measured the serum 25(OH)D in 5109 pregnant Australian women in the first trimester and concluded that low serum 25(OH)D during pregnancy was not associated with adverse pregnancy outcomes, including SGA^13^. In addition, a cohort study involving 2382 mother-child pairs did not find any evidence of an association between maternal circulating 25(OH)D and BW, birth length and risk of SGA^18^. To the best of our knowledge, only an observational study among 79 newborns conducted in Turkey and our previous study have reported an increased risk of macrosomia for pregnant woman with low 25(OH)D concentrations^19,22^. Therefore, well-designed studies conducted in multiple centres and adequately powered randomized controlled trials for maternal vitamin D supplementation are needed.

In our previous *in vitro* and *in vivo* study, we concluded that vitamin D deficiency during pregnancy may promote the proliferation and differentiation of pre-adipocytes, which may be associated with the methylation alterations of genes, such as *Vldlr* and *Hif1α*, ultimately leading to offspring obesity^23^. Moreover, it was reported that serum 25(OH)D <50 nmol/L was significantly associated with new-onset obesity^24^. Recently, Wang *et al*. performed a genome-wide association study (GWAS) of the gut microbiota and discovered a significant association of the VDR gene (encoding vitamin D receptor) with gut microbial characteristics, which is essential for bile acid and fatty acid metabolism^25^. Even so, understanding the role of maternal vitamin D status in offspring outcomes merits further exploration.

There are some limitations to this study. This is a cross-sectional study, and thus it is not possible to determine a causal relationship between vitamin D deficiency and macrosomia. Moreover, as data for prenatal weight were unavailable, the intrapartum BMI was adjusted for statistical analysis. Although the intrapartum BMI was closely related to BW, the predictive value of intrapartum BMI for macrosomia is limited because of late testing. In addition, other factors influencing BW were not considered, such as outdoor activities, dietary intake, gestational weight gain and genetic factors, which may contribute to the residual confounders in our study. Further prospective studies considering the above potential confounders and incorporating diverse populations with long-term effects are warranted and would have important implications for public health policy. Nonetheless, our study has provided robust epidemiological evidence that low serum 25(OH)D in pregnant women was significantly associated with an increased risk of macrosomia. The findings suggested that vitamin D supplements in pregnancy should be encouraged to prevent macrosomia.

## Materials and Methods

This study was conducted according to the guidelines in the Declaration of Helsinki and all procedures involving human subjects were approved by the Institutional Review Board of Nanjing Maternity and Child Health Care Institute. This trial is registered at ClinicalTrials.gov with clinical trial identifier number NCT02236221.

### Participants and study design

We conducted a nested case-control study in a cohort of 4718 women. All women who had attended second- and third-trimester pregnancy complication screenings and subsequently delivered at Nanjing Maternity and Child Health Care Hospital, between March 2012 and February 2015, were eligible. Written informed consent was obtained from all participants. Fasting blood samples were collected for routine multiple marker screenings, and serum aliquots were stored at −80 °C. Maternal information for archived serum samples were derived from the laboratory database and the corresponding birth outcomes were obtained via electronic medical record collection and information extraction. The extracted variables included maternal age (in year), birthplace (Jiangsu province or other provinces), intrapartum BMI (kg/m^2^), gestational weeks at birth, fetus gender, birth weight, status of gestational diabetes (fasting glucose concentration ≥5.5 mmol/L or 2-h plasma glucose concentration ≥8.0 mmol/L), parity (nulliparae or multiparae), sampling trimester (second or third), abnormal pregnancy history and sampling season. The pregnant women with previously diagnosed hypertension (chronic or pregnancy) or diabetes (pre-gestational or gestational), kidney disease, uterine fibroids, multiple gestation or any other significant pre-existing chronic medical disease were excluded.

From the total cohort of 4718 women, 545 women subsequently delivered infant with macrosomia, with birth weight ≥4000 g and had met all of the above inclusion and exclusion criteria. These cases were matched by maternal age and birthplace, at a 1:2 ratio, to a random computer-generated reference group of 1090 women who delivered neonate of normal weight (2500 g ≤ birth weight <4000 g), using the same inclusion and exclusion criteria.

### Vitamin D measurement

The maternal serum concentrations of 25(OH)D were measured by using an *in vitro* diagnostic enzyme immunoassay kit, OCTEIA 25-Hydroxy Vitamin D (Immunodiagnostic Systems, Boldon, United Kingdom), according to the manufacturer’s instructions. The inter- and intra-assay coefficients of variation were 5.1% and 4.8%, respectively. Blank (water) controls in each plate were used for quality control and more than 5% of the samples were randomly selected to repeat. The reported analytic sensitivity of the immunoassay was 6.8–380 nmol/L. Commonly used cutoffs to define 25(OH)D status were assigned at 25, 37.5, 50 and 75 nmol/L.

### Statistical analysis

Differences in the maternal characteristics and 25(OH)D serum concentrations between women who delivered macrosomia and controls were calculated by the Student’s *t*-test (for continuous variables), *χ*^2^ test (for categorical variables) and Mann-Whitney test (for 25(OH)D concentrations). Logistic regression analysis was performed to assess the crude and adjusted associations between 25(OH)D concentrations (<25.0, 25.0–37.4, 37.5–49.9, <50.0, >75.0 nmol/L vs. 50.0–74.9 nmol/L) and macrosomia risk by computing the odds ratios (OR) and their 95% confidence intervals (CIs). In the multivariate regression analysis, maternal age, birthplace, intrapartum BMI, gestational weeks at birth, fetus gender, status of gestational diabetes, parity, sampling trimester, abnormal pregnancy history and sampling season were examined. The relationship between 25(OH)D concentrations and the risk of macrosomia was explored by the smoothing plot.

A risk prediction model to classify women who delivered macrosomia and controls was constructed according to the following steps^26^: (1) Prediction factor selection: maternal age, birthplace, intrapartum BMI, gestational weeks at birth, fetus gender, status of gestational diabetes, parity, sampling trimester, abnormal pregnancy history, sampling season and 25(OH)D deficiency (<50.0 nmol/L) were considered predictive factors by conducting a stepwise logistic regression. (2) Risk model construction: the variables that remained in the stepwise model were included, and the risk prediction model was constructed using a logistic regression model. (3) Risk model evaluation: the model performance was evaluated by conducting a receiver-operator characteristic curve analysis, and the area under the curve was used to classify the women who delivered macrosomia and controls. The model’s calibration was assessed by Hosmer-Lemeshow *χ*^2^ test. A graphical nomogram was also produced for the model so that the individual-specific probabilities of delivering macrosomia could be easily approximated. All statistical analyses were performed with the R software (version 2.13.0), and *P* ≤ 0.05 in a two-sided test was considered statistically significant.
